# Hepatitis B surface antigen variants in voluntary blood donors in Nanjing, China

**DOI:** 10.1186/1743-422X-9-82

**Published:** 2012-04-14

**Authors:** Yang Yong-lin, Fu Qiang, Zhang Ming-shun, Cai Jie, Ma Gui-ming, Huang Zu-hu, Cai Xu-bing

**Affiliations:** 1 Nanjing Red Cross Blood Center, Nanjing, China; 2 Department of Infectious Disease, the First Affiliated Hospital of Nanjing Medical University, Nanjing, China; 3 Department of Microbiology and immunology, Nanjing Medical University, Nanjing, China

**Keywords:** Hepatitis B virus, Hepatitis B surface antigen, Mutation, Genotype

## Abstract

**Background:**

Hepatitis B virus (HBV) is still one of the serious infectious risks for the blood transfusion safety in China. One plausible reason is the emergence of the variants in the major antigenic alpha determinant within the major hydrophilic region (MHR) of hepatitis B surface antigen (HBsAg), which have been assumed to evade the immune surveillance and pose a challenge to the disease diagnosis. It is well documented that some commercial ELISA kits could detect the wild-type but not the mutant viruses. The high prevalence of HBV in China also impaired the application of nucleic acid testing (NAT) in the improvement of blood security. Molecular epidemiological study of HBsAg variations in China is still limited. This study was designed to identify the prevalence of mutations in the HBsAg in voluntary blood donors in Nanjing, China.

**Methods:**

A total of 20,326 blood units were enrolled in this study, 39 donors were positive for HBV S gene in the nested-PCR. Mutations in the major hydrophilic region (MHR; aa 99-169) were identified by direct sequencing of S region.

**Results:**

Among of 20,326 blood units in the Red Cross Transfusion Center of Nanjing from October 2008 to April 2009, 296 samples (1.46%, 296/20,326) were HBsAg positive in the 2 successive rounds of the ELISA test. In these HBsAg positive units, HBV S gene could be successfully amplified from 39 donors (13.18%, 39/296) in the nested-PCR. Sequence analysis revealed that 32 strains (82.1%, 32/39) belong to genotype B, 7 strains (17.9%, 7/39) to genotype C. Besides well known G145R, widely dispersed variations in the MHR of S region, were observed in 20 samples of all the strains sequenced.

**Conclusions:**

HBV/B and HBV/C are dominant in Nanjing, China. The mutations in the MHR of HBsAg associated with disease diagnosis are common.

## Introduction

Hepatitis B is a major global health problem and the most serious type of viral hepatitis, leading to the liver cirrhosis and liver cancer in the chronic consequences. Worldwide, it is estimated that more than 2 billion people have been infected with Hepatitis B virus (HBV), one-third reside in China [1]. Over the last 20 years, although the vaccination program has contributed to a reduction in HBV infection, decreasing from nearly 10% to approximately 7% in the general population [2], China is still an intermediate/high endemic country for hepatitis [3,4].

HBV is still one of the serious infectious risks for the blood transfusion safety in China [5-8]. One plausible reason is the emergence of the variants in the major antigenic alpha determinant of hepatitis B surface antigen (HBsAg) [9-17], which have been assumed to evade the immune surveillance and pose a challenge to the disease diagnosis [18-21]. It is well documented that some commercial ELISA kits could detect the wild-type but not the mutant viruses [5,22]. HBV nucleic acid testing (NAT) are introduced as a mandatory test for the blood supply to reduce such a risk in some developed countries [23-28]. The high prevalence of HBV in China, however, impaired the application of NAT in the improvement of blood security. Moreover, HBV NAT is procedurally cumbersome and incurs high costs [29].

In China, an effective strategy based on the screen of HBsAg has been adopted and observed strictly by blood centers to reduce the risk of HBV transmission through blood transfusion. Before the donation in the street, all of the volunteers were asked for a fast colloidal gold assay for HBsAg. Only the HBsAg negative volunteers were accepted for blood donation. And the blood units in the centers were further monitored for HBsAg with 2 successive rounds of the commercial ELISA kits. Few blood units, if positive in either or both ELISA test, which is more sensitive than the fast colloidal gold assay, would be discarded. However, it could not rule out that ELISA occasionally fails to detect HBV-infected donors, partially due to the variants of HBsAg, especially the alpha determinant region located at aa124-aa147 within the major hydrophilic region (MHR) from aa99 to aa169 [30,31].

Variants of HBsAg in China have been reported elsewhere. The alpha determinant mutation seemed to be uncommon in community-based population of Shandong province and the mutation sites were relatively scattered [32]. G145R was the major variation in the HBV isolates responsible for the occult HBV infections in Xiamen, China [33]. In the blood donors with occult HBV infection (OBI) in Nanjing, mutation was observed in the alpha determinant of HBsAg from only 1 of 5 samples [34]. However, the samples enrolled in those experiments were rather limited. And more importantly, the blood donors with OBI, which is defined as the 'presence of HBV DNA in the liver of individuals testing HBsAg-negative with currently available assays' [35], is different from the blood donors with HBsAg positive enrolled in this study. Here, we have analyzed HBV genotype/serotype and mutations in MHR of HBsAg from 39 HBV S gene positive samples from 20,326 voluntary blood donors in Nanjing, which is the capital of Jiangsu Province and the home for over 8 million people.

## Methods

### Samples

From October 2008 to April 2009, a total of 20,326 blood units were collected by the Red Cross Transfusion Center of Nanjing. The subjects in the study ranged from 20 to 55 years old (mean ± SD; 32.3 ± 10.5), with 10,690 males (52.5%) and 9,636 females (47.4%). All of the samples were HBsAg negative upon a commercial colloidal gold immunochromatography assay (InTec Ltd, Xiamen, China) before the blood donation in the street. Approval from the local institutional ethics committee was obtained before the study.

### HBV ELISA

The blood units in the center were further detected for HBsAg with two different commercial ELISA kits, from InTec Ltd, Xiamen and KHB Ltd, Shanghai. All assays were performed according to the manufacturer's instructions. Blood samples were separated and stored at -70°C until use.

### Sequencing of the S gene

All samples that were HBsAg-positive by either ELISA were subjected to nested PCR targeting the S gene. Viral DNA was extracted from 400 ul of plasma using a QIAamp DNA Blood Mini Kit (QIAGEN, Hilden, Germany). The first round of PCR was performed using an outer primer set (5'- ACTGTCTCTGCCATATCGTCA-3', 5'-CCAACACCCAATTACATATC-3') for 30 cycles (94°C for 30 s, 56°C for 30 s, and 72°C for 40 s). The second round was performed using an inner primer set (5'- ATGGAGAACATCGCATCAGG-3', 5'-TTAAATGTATACCCAAAGAC-3') for 35 cycles (94°C for 30 s, 58°C for 30 s, and 72°C for 30 s). PCR products were sequenced directly on an ABI Prism 3130X automatic genetic analyzer (Invitrogen, Shanghai).

## Results

### HBV prevalence in the blood donors

Among of 20,326 blood units, 296 samples (1.46%, 296/20,326) were HBsAg positive in the 2 successive rounds of the ELISA test, with 189 males (63.9%) and 107 females (36.1%). There were 10 samples positive for InTec Ltd only, 9 samples positive for KHB Ltd only, and 277 samples positive for both. In these 296 HBsAg positive units, HBV S gene could be successfully amplified from 39 donors (13.18%, 39/296). Sample 502019, 509344 and 573590 were negative for InTec ELISA but positive for KHB ELISA, sample 506591 was positive for InTec ELISA but negative for KHB ELISA. Table 1 summarizes the ELISA results of these 39 donors.

**Table 1:** The ELISA results of 39 HBV S gene DNA positive donors

sample	InTec	KHB	sample	InTec	KHB	sample	InTec	KHB
502019^#^	0.047	0.138	522801	1.885	1.285	561451	2.542	9.991
502800	9.993	9.998	525706	0.387	0.517	563060	1.653	1.309
505194	9.994	9.994	526765	0.107	0.082	564014	2.928	9.985
506519^&^	0.18	0.01	530119	1.288	1.103	565200	1.103	1.303
506588	2.963	9.999	531355	2.498	2.711	565380	2.691	2.785
509344^#^	0.027	2.111	531508	0.127	0.234	566102	0.114	0.12
510260	2.855	2.169	532014	2.456	1.743	572640	1.116	1.232
510351	9.991	9.993	534991	9.999	9.999	573590^#^	0.001	2.827
513357	2.263	9.992	546227	0.272	0.26	574273	2.834	9.999
517437	0.052	0.088	549409	2.615	2.745	577612	10.001	9.992
518796	2.443	2.922	554464	0.235	0.426	578193	2.83	2.454
519821	0.258	0.627	560744	9.992	2.648	578265	0.316	0.538
522082	0.134	0.161	561321	2.807	9.993	579524	2.755	9.999

1. The cutoff value of was 0.105, which was calculated from the twice that of mean OD of the negative control plus 0.005. The mean OD of the negative control is taken as least as 0.050

2. # indicate the sample was positive for KHB ELISA only (502019, 509344 and 573590)

3. & indicate the sample was positive for InTec ELISA only (506591)

### HBV genotypes and serotypes

**Figure 1:**
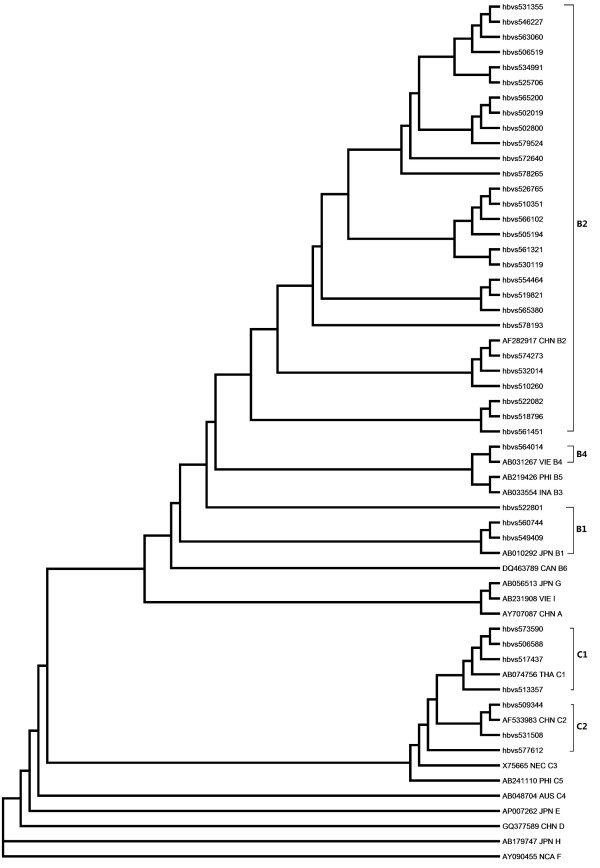
**Phylogenetic analysis based on nucleotide sequencing of the small S region**. The tree contains sequences from 39 samples and a set of representative sequences belonging to genotypes and subgenotypes retrieved from Genebank. The phylogenetic tree was constructed using the neighbor-joining method in ClustalW2.

Phylogenetic analysis of the S gene sequences of the 39 cases indicated that 32 strains (82.1%, 32/39) belonged to genotype B, 7 strains (17.9%, 7/39) to genotype C. No other genotypes were observed. To define the subgentoype of HBV, reference strains of B1 ~ B6 and C1 ~ C4 retrieved from Genebank were also included in the analysis. Among of strains from genotype B, 28 strains (87.5%) were from B2, 3 strains from B1, only 1 strain (564014) was from B4. Seven strains from genotype C were divided into four C1 and three C2 (Figure 1).

**Figure 2:**
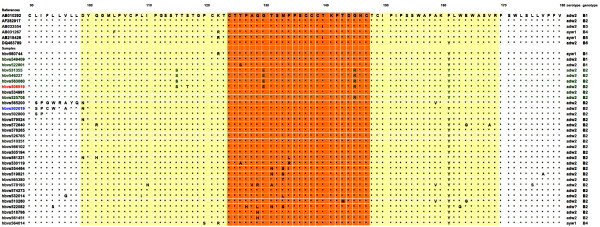
**Multiple sequence alignment with hierarchical clustering based on the protein sequences of the MHR of HBsAg from genotype B**. Yellow shaded area indicated the MHR (aa99-aa169), Red shaded area indicated the alpha determinant region (aa124-aa147). Sample 506519 and 502019 was HBsAg negative in one of the kit from KHB Ltd or InTec Ltd.

**Figure 3:**
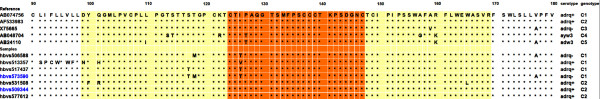
**Multiple sequence alignment with hierarchical clustering based on the protein sequences of the MHR of HBsAg from genotype C**. Yellow shaded area indicated the MHR (aa99-aa169), Red shaded area indicated the alpha determinant region (aa124-aa147). Sample 573590 and 509344 was HBsAg negative in one of the kit from KHB Ltd or InTec Ltd.

In the genotype B group, 29 sequences belonged to serotype adw2 (122 K + 160 K + 127P), samples 564014 and 560744 were from ayw1 (122R + 160 K + 127P + 159A), but the sample 522082 (122 K + 160 K + 127H) could not be classified into serotype adw2, adw3 or adw4 for the mutation P127H. Seven strains from genotype C group were divided into five adrq + (122 K + 160R + 177 V) and two adrq- (122 K + 160R + 177A) (Figures 2 and 3).

### Mutations in MHR of HBsAg

To explore the mutations affecting the antigenicity of HBsAg, MHR within the major antigenic alpha determinant were analyzed. In 32 sequences of genotype B cases, there were 21 samples (65.6%, 21/32) with multiple mutations in the major hydrophilic region. The well-known G145R substitution was found in the 5 sequences, i.e., 531355, 546227, 563060, 506519, 525706, all of which were from B2/adw2. Besides G145R, there were different types of aa substitutions in the MHR that were associated with lower reactivity in HBsAg assays reported in previous studies: i.e. D99N, Q101R/H, L109I, I110N, T115S, S117T, P120S, K122R, S126T/A, P127H, A128V, Q129R/H/L, G130E, T131N/A, M133T/L/S, F134R/I/L, T143M, A159V, F161Y, W163G, E164G, V168A. In the sequences 502019 and 565200, mutations were clustered before but not within the MHR: L91S, I92P, F93C/G, L94W, L95R, V96A, L97Y, and L98Q. In 7 sequences of genotype C cases, mutations in the MHR could be found in all of the samples, i.e. D99N, Y100F, Q101R/H, L110I, T113S, S114T, S117T, T118M, I126T/V, W165L, and V168A, which may be associated with antigenicity of HBsAg. G145R substitution, however, was not observed. Similar with sequences 502019 and 565200 from genotype B, sequence 513357 from genotype C showed mutations clustered before the MHR: L91S, I92P, F93C, L94W, V96W, and L97F.

No mutation was observed with C residues at position 124, 137, 139, 147, or 149, which proved to play a critical role in the conformational structure of the alpha determinant in vitro studies. Phe at position 93 was replaced with cysteine residue (F93C) in the sequence 502019 from genotype B and sequence 513357 from genotype C.

## Discussion

In the HBsAg screening test, whether NAT should be incorporated into donor screening in China is still in debate, although NAT could reduce the risk of HBV transmission through blood donation. One of the reason is that NAT is cubersmome. Thus, before the NAT incorporated into donor screening in China, standard operation procedures should be made and observed in the blood banks.

In China, the fast colloidal gold assay is the fist defence line of blood safety in China. Compared with the fast colloidal gold assay, ELISA is more sensitive. And significant few samples (1.46%, 296/20,326) were positive in the ELSIA but negative in the street assay before the blood donation. Two different commercial HBsAg kits from InTec Ltd, Xiamen and KHB Ltd, Shanghai are widely applied in China. It should be noted that some samples were only positive for one of them but not both, which suggested that these two kits may be complementary. In these 296 HBsAg positive units, HBV S gene could be successfully amplified from 39 donors (13.18%, 39/296), which confirmed the ELISA assay and proved that at least these samples were contagious and should be discarded. Indeed, all of the HBsAg positive samples were considered to be dangerous and could not be used in the blood transfusion. The failure of PCR targeting S gene in the majority of HBsAg positive samples was more likely due to the low virus load in these samples.

China is a vast country and different genotypes of HBV have been reported. In Shenyang, the largest city in the northeast of China, HBV/C was widely distributed (50%) in chronic patients or asymptomatic carriers along with HBV/B (22%) and HBV/D (28%) [36]. In Xian from the northwest region of China, 11 strains from blood donors were clustered into genotype B and C [30]. In Guangzhou, the largest city in southern China, 6/7 of occult HBV infection (OBI) strains from blood donors were from HBV/C [37]. In Qidong, the most eastern city in China, all of the virus isolates from 81 vaccinated young adults with OBI were also genotype C [38]. Compared with Guangzhou and Qidong, all of five blood donors with OBI in Nanjing were infected with HBV/B [34]. Consistent with the findings in the blood donors with OBI in Nanjing [34], the present study demonstrates that HBV/B is also dominant (82.1%, 32/39) in the blood donors with HBsAg positive in Nanjing.

Given the diversity of HBV genotypes [39], the classification of a novel HBsAg amino acid change as a mutant should be contingent on a substantial alteration in viral function, such as antigenicity, infectivity, replication, and morphology, which is attributable to the specific change. The major hydrophilic region (MHR) extending from aa99 to aa169 clustered with a highly conformational epitope is critical to the antigenicity of HBsAg [30,31]. Thus, amino acid substitution in the MHR, either from variants in natural isolates or mutants under immunological pressure, could cause the mistaken diagnosis of HBV in the HBsAg screening test.

Amino acid changes of HBsAg in China have been reported elsewhere, focusing on the chronic carrier [11], OBI patients [33,34], vaccinated people [40]. The variants of HBV on the general population in China [32], however, remain elusive. In Shandong province, the alpha determinant variation seemed to be uncommon (14.7%, 15/102) in the community-based population and the mutation sites were relatively scattered [32]. In the present study, the variations with the alpha determinant aa124-aa147 were usual. In the 32 strains from genotype B, aa substitutions could be observed in 16 sequences; in the 7 HBV/C strains, variations could be identified in 4 sequences. Totally, the mutation ratio was over 50% (51.3%, 20/39), which was much higher than in Shandong Province, reflecting the more widely application of HBV vaccine in Nanjing [19].

The well-known G145R was the major variation in the HBV isolates responsible for the occult HBV infections in Xiamen, China [33]. G145R also has a direct impact on the diagnosis [41]. Here, G145R could be identified in 5 samples, i.e., 531355, 546227, 563060, 506519, 525706. Among of them, 506519 was negative in the HBsAg ELISA kit from KHB Ltd, Shanghai, but positive in the kit from InTec Ltd, Xiamen. The remaining four samples (531355, 546227, 563060 and 525706) were positive for either of two kits. Since the mutation pattern in the MHR of 531355, 546227 and 563060 was very similar with 506519, variation outside of MHR may also play important roles for the loss binding of 506519 in the HBsAg ELISA kit from KHB Ltd. Sample 509344 was negative in the HBsAg ELISA kit from InTec Ltd, Xiamen. However, no mutation could be identified within the MHR, further suggesting the critical roles of domains outside of MHR in the antigenicity of HBsAg. Besides of G145R, different aa substitutions within and outside of MHR were also found in the samples from HBV/B and HBV/C. The effects of these variations on the HBsAg and HBV warrants further investment.

## Conclusion

HBV/B and HBV/C are prevalent in the blood donors with HBsAg positive, consistent with the previous finding in the OBI patients in Nanjing. Mutations in the MHR which may associate with HBsAg diagnosis are common.

## Abbreviations

HBV: Hepatitis B virus; HBsAg: Hepatitis B surface antigen; ELISA: Enzyme-linked immunosorbent assay; NAT: Nucleic acid testing; MHR: Major hydrophilic region; PCR: Polymerase chain reaction; OBI: Occult HBV infection

## Competing interests

The authors declare that they have no competing interests.

## Authors' contributions

YYL contributed in data analysis and participated in manuscript preparation. FQ participated in the ELISA test analysis. ZMS drafted the manuscript. CJ participated in the nested-PCR. MGM performed molecular genetic studies. CXB coordinated the research effort. HZH conceived of the study, participated in its design and coordination. All authors have read and approved the final manuscript.
